# Interrater and intrarater reliability of photoplethysmography for measuring toe blood pressure and toe-brachial index in people with diabetes mellitus

**DOI:** 10.1186/1757-1146-5-13

**Published:** 2012-06-07

**Authors:** Christopher Scanlon, Kris Park, David Mapletoft, Lindy Begg, Joshua Burns

**Affiliations:** 1 Diabetes Service, Nepean Hospital, Nepean Blue Mountains Local Health Network, Level 3 West Block, Derby St Kingswood, NSW, 2747, Australia; 2 Foot Wound Clinic, Department of Surgery, Westmead Hospital, Level 1 Block E Hawkesbury Rd, Westmead, NSW, Australia; 3 Arthritis and Musculoskeletal Research Group, The University of Sydney / Institute for Neuroscience and Muscle Research and Paediatric Gait Analysis Service of NSW, The Children’s Hospital at Westmead, Sydney, NSW, 2145, Australia

**Keywords:** Toe brachial index, Photoplethysmography, Peripheral arterial disease, Ankle brachial index, Toe pressures, Doppler, Diabetes mellitus

## Abstract

**Background:**

A reliable tool to measure arterial flow to the feet in people with diabetes is required given that they are particularly prone to peripheral arterial disease. Traditionally, the ankle brachial index (ABI) has been used to measure arterial circulation, but its application is limited due to calcification of larger arteries. More recently, toe pressure and the toe brachial index (TBI) has been suggested as superior to ABI measurements because they assess smaller digital arteries less prone to arterial calcification. However, reliability studies for the clinical use of photoplethysmography (PPG) in people with diabetes are lacking.

**Methods:**

Sixty people with diabetes mellitus (35 males and 25 females, mean age 59.6 yrs) consented to take part in the study. The majority (92%) had type 2 diabetes and 8% had type 1 diabetes. Forty-three percent were diagnosed as having peripheral neuropathy when tested using a biothesiometer and 15% were current smokers (10 – 40/day). A podiatrist and a diabetes educator measured toe and brachial blood pressure independently and in a random order using PPG. These measurements were repeated again seven days later, and subsequently analysed with intraclass correlation coefficients (ICC), 95% confidence intervals (CI) and standard error of measurement (SEM).

**Results:**

The intrarater reliability of measuring toe pressures was excellent (ICC_3,1 =_0.78-0.79, SEM 8 mmHg) and interrater reliability was also excellent (ICC_2,2_ = 0.93, SEM 4 mmHg). The intrarater reliability for measuring brachial pressures was generally poor (ICC_3,1_ = 0.40 – 0.42, SEM 19 mmHg) and interrater reliability was fair-good (ICC_2,2_. 0.65, SEM 14 mmHg). The TBI intrarater reliability was fair-good (ICC_3,1_ = 0.51-0.72, SEM 0.08), whilst the interrater reliability of TBI was excellent (ICC_2,2_ = 0.85, SEM 0.07).

**Conclusions:**

Based on these results, interrater and intrarater reliability of PPG is excellent for measuring toe blood pressure, good for TBI and only fair for brachial pressures in people with diabetes mellitus.

## Background

Peripheral arterial disease (PAD) is one of the most serious complications affecting the lower limbs of people diagnosed with diabetes mellitus. PAD is an indicator for widespread atherosclerosis and increased mortality rates [1]. It is imperative that PAD is adequately assessed and diagnosed early in patients with diabetes to offer patients the best possible outcomes, and prevent foot ulceration and subsequent foot or lower limb amputation. Arterial flow to the periphery has traditionally been measured by palpation of dorsalis pedis and posterior tibial pulses or through the calculation of the ankle brachial index (ABI) [2-5]. The ABI is a ratio of the systolic ankle blood pressure and the systolic brachial blood pressure. It is generally accepted that a ratio of 1:1 for the ABI suggests adequate peripheral perfusion. However, both of these tests have their limitations. Palpation of pedal pulses is a subjective measure and pulses become increasingly difficult to palpate as PAD becomes more significant [6-8]. There is also large interrater variability in palpation of pedal pulses with inexperienced clinicians [9,10]. The ABI is a reliable measure of PAD in patients without diabetes and its sensitivity and specificity are excellent. However, for those patients with long standing, or poorly controlled diabetes the ABI has limited application due to the likelihood of falsely elevated readings. This occurs due to the non compressibility of the larger calcified arteries that is a complication of long standing diabetes [8,11-13].

Finding a reliable test to measure the perfusion distal to the ankle is required to adequately assess and treat patients with diabetes and PAD. Damage to the smaller branches of the arteries, known as microvascular disease, is common in diabetes. In particular it is important to understand the severity of microvascular disease distal to the ankle in patients who have active foot ulceration, as it provides the clinician with a greater understanding of healing potential and whether there is an opportunity for the vascular team to improve the flow to the extremity via revascularisation techniques such as angioplasty, arterial stenting or bypass surgery. The ABI is unable to adequately assess these microvascular complications as it measures proximal to the ankle joint encompassing the arterial flow of the anterior and posterior tibial arteries and does not identify any occlusion or calcification of vessels distal to this site [13,14]. The concept of measuring toe blood pressures and calculating the toe brachial index (TBI) is not new [7]. The measurement of toe pressures using a PPG unit such as the Hadeco Smartdop is a relatively simple procedure involving a small digital toe blood pressure cuff and PPG probe to capture red blood cells as they pass through the underlying tissue. To calculate the TBI, the process is the same as calculation of the ABI, with the exception of the ankle pressure being replaced by the toe pressure. One study reported quite high intra-class correlation coefficients using the Hadeco Smartdop 45 PPG to assess systolic toe pressures and TBI in people with diabetes, but with a wide range of error [15]. These reliability concerns may relate to the type of PPG unit evaluated which required manual sphygmomanometer to assess systolic toe pressures and TBI, or may relate to the small sample size of 30 participants. Therefore the aim of this study was to evaluate intrarater and interrater reliability of measuring systolic toe pressures and TBI using the Hadeco Smartdop 30EX PPG with built-in automated cuff inflator in 60 patients with diabetes mellitus.

## Methods

### Participants

A sample of 60 participants with diabetes mellitus confirmed by an oral glucose tolerance test participated in this study. All participants were recruited through advertisements at the Diabetes Service and High Risk Foot Clinic at Nepean Hospital, and the Foot Wound Clinic at Westmead Hospital. Participants were excluded if they were under 18 years, had an active ulceration on one of their great toes, or had recent history of infection, osteomyelitis or acute charcot neuroarthropathy. The study was approved by the human research and ethics committee of Nepean Hospital (HREC no: 08/045).

### Instrument

The device used to obtain the pressure measurements was the Hadeco Smartdop 30EX PPG. This particular unit was chosen as it is widely used in our high risk foot clinics and associated teaching facilities. Two studies have used the Hadeco Smartdop 30EX PPG as an effective screening test and outcome measure of coronary and peripheral arterial disease [16,17] . This device has the ability to measure and calculate ankle, brachial, toe and segmental leg blood pressures as well as PPG and doppler waveforms. It has a built-in automated cuff inflator and has the ability to change cuff sizes. Blood pressures can be obtained by using either the doppler or PPG probe to measure the blood flow during occlusion. The device is a portable unit that runs off battery or mains power.

### Procedure

The participants were measured by two assessors: assessor 1 (CS) was a podiatrist with three years clinical experience and two years experience using the PPG; assessor 2 (DM) was a registered nurse and diabetes educator with over 30 years clinical experience, and only four hours training in the use of the PPG.

All participants were required to lay supine for five minutes prior to any assessment being conducted. After this time assessor 1 conducted a standard neurological and vascular foot assessment to determine an overall picture of the participant’s current foot health. Toe and brachial pressures were assessed on a randomised limb by a randomised first assessor, with results blinded between assessor 1 and 2. Brachial pressures were obtained using the automated blood pressure cuff attached to the Hadeco Smartdop 30EX unit and the 8mhz doppler probe. Toe pressures were obtained using the automated toe blood pressure cuff in conjunction with the PPG probe attached to the Smartdop 30EX unit. Toe pressures and the TBI were automatically calculated by the unit and a printout was obtained. Each participant returned seven days after their first assessment for the final measures to be collected. Participants were required to advise the assessors if anything had changed with their medical history, including changes to current medications, alteration in smoking habits between visits.

### Statistical methods

Descriptive statistics were calculated to characterise the study sample in SPSS version 18.0 (Chicago, IL). Normality of data distribution was assessed with the Kolmogorov-Smirnov test with Lilliefors significance correction and the appropriate parametric or non-parametric test was subsequently applied. Intraclass correlation coefficients (ICC) and 95% confidence intervals (CI) were calculated to determine intrarater reliability (ICC_3,1_) and interrater reliability (ICC_2,2_) [18]. Benchmarks suggested by Fleiss were used to interpret ICC values, where a value of 0.75 or greater indicates excellent reliability; 0.40 to 0.75, fair to good reliability; and 0.40 or less poor reliability [19]. To determine the absolute between-trial variability in scores, the standard error of measurement (SEM) was calculated [18].

## Results

Demographics and physical characteristics of all participants are shown in Table 1. Intrarater and interrater reliability results are presented in Tables 2 and 3, and summarised below.

**Table 1:** Demographic details and physical characteristics of the 60 participants

**Variable**	**Total Participants (*N*** **= 60)**
Age, *mean yrs (SD), range*	59.6 (10.5), 23 - 80
Gender, *male (%)*	35 (58)
Body mass index, *mean kg/m*^*2*^*(SD), range*	32.1 (5.9), 17.1 - 45.9
Diabetes mellitus, *no. (%)*	
Type 1	5 (8)
Type 2	55 (92)
Duration of diabetes, *median yrs (25th and 75th centile), range*	9 (4 and 16), 1 - 44
*HbA1c, median (%) (25th and 75th centile), range*	7.3 (6.6 and 8.1), 5.8 - 12
Current smoker, *no. (%)*	9 (15)

**Table 2:** Intrarater reliability of PPG for measuring toe blood pressure and TBI in 60 people with diabetes mellitus

**Variable**	**Assessor**	**Session 1 Mean (SD)**	**Session 2 Mean (SD)**	**ICC**_**3,1**_	**95 % CI**	**SEM**
Toe pressure*, mmHg*	1	98.7 (29.3)	103.3 (24.3)	0.78	0.65 – 0.86	8.5
Toe pressure*, mmHg*	2	102.1 (27.3)	97.5 (23.5)	0.79	0.67 – 0.87	7.6
Brachial pressure*, mmHg*	1	130.7 (24.2)	129.3 (23.3)	0.42	0.19 – 0.61	19.5
Brachial pressure*, mmHg*	2	129.1 (22.2)	127.7 (23.4)	0.40	0.17 – 0.59	19.4
TBI	1	0.78 (0.26)	0.81 (0.19)	0.51	0.30 – 0.68	0.16
TBI	2	0.81 (0.24)	0.76 (0.18)	0.72	0.57 – 0.82	0.08

**Table 3:** Interrater reliability of PPG for measuring toe blood pressure and TBI in 60 people with diabetes mellitus

**Variable**	**Assessor 1 Mean (SD)**	**Assessor 2 Mean (SD)**	**ICC**_**2,2**_	**95% CI**	**SEM**
Toe pressure*, mmHg*	98.7 (29.3)	102.1 (27.3)	0.93	0.88 – 0.96	4
Brachial pressure*, mmHg*	130.7 (24.2)	129.1 (22.2)	0.66	0.42 – 0.79	14
TBI	0.78 (0.26)	0.81 (0.24)	0.85	0.79 – 0.91	0.07

### Intrarater reliability

#### Toe pressures

The intrarater ICC for assessor 1 (CS) was excellent (ICC_3,1_ = 0.78, 95% CI 0.65 - 0.86) and the measurement error was 8.5 mmHg. The intrarater ICC for assessor 2 (DM) was excellent (ICC_3,1_ = 0.79, 95% CI 0.67 - 0.87) and the measurement error was 7.6 mmHg.

#### Brachial pressures

The intrarater ICC for assessor 1 (CS) was fair (ICC_3,1_ = 0.42, 95% CI 0.19 - 0.61) and the measurement error was 19 mmHg. The intrarater ICC for assessor 2 (DM) was poor (ICC_3,1_ = 0.40, 95% CI 0.17 - 0.59) and the measurement error was 19 mmHg.

#### TBI

The intrarater ICC for assessor 1 (CS) was fair (ICC_3,1_ = 0.51, 95% CI 0.30 - 0.68) and the measurement error was 0.16. The intrarater ICC for assessor 2 (DM) was good (ICC_3,1_ = 0.72, 95% CI 0.57 - 0.82) and the measurement error was 0.08.

### Interrater reliability

#### Toe pressure

The interrater ICC was excellent (ICC_2,2_ = 0.93, 95% CI = 0.89 - 0.96) and the measurement error was 4 mmHg (Table 3).

#### Brachial pressure

The interrater ICC was good (ICC_2,2_ = 0.65, 95% CI = 0.42 - 0.79) and the measurement error was 14 mmHg.

#### TBI

The interrater ICC was excellent (ICC_2,2_ = 0.85, 95% CI = 0.74 - 0.91) and the measurement error was 0.07.

## Discussion

Overall our results showed that the intrarater and interrater reliability of Hadeco Smartdop 30EX PPG was excellent for measuring toe blood pressure, good for TBI and poor for brachial pressures in 60 adults with diabetes mellitus. This finding supports previous research recommending PPG as the most effective and reliable measure of systolic toe pressures in the general community [2,14,20,21].

Systolic toe pressures using PPG give a quantifiable value of the arterial supply to the distal foot. For clinicians working with the high risk foot it is important to determine the healing potential of a patient with diabetes, and systolic toe pressures allow us to do this. They are a simple, yet very effective component of a standard vascular foot assessment. Our results enable better adherence to the suggested threshold of <30 mmHg systolic toe blood pressure, which infers ischaemia, decreased healing potential and increased amputation risk, to ensure more appropriate referral to vascular specialists [12,21-23]. After obtaining toe pressure results the clinician can then decide on the appropriateness of a referral for additional arterial studies or make evidenced-based decisions on the most appropriate treatment plan for the patient, such as vascular intervention, wound dressings or sharps debridement. The TBI is the ratio between the toe systolic pressure and the brachial systolic pressure, similar to the ABI ratio. When using the TBI as an indicator of peripheral arterial supply the clinician can use the reference ranges that have been reported by multiple authors to determine the level of PAD in patients with and without diabetes. Many authors [12,21-23] have assigned a TBI <0.64 indicative of arterial insufficiency, whilst others have suggested a TBI < 0.75 is a clinically significant sign of arterial disease [8].

Reliable health-related outcome measures provide the clinician with robust markers of disease severity. As such there are a number of clinical implications from the results of this study. Toe pressures and the TBI can be used as an adjunct to a standard peripheral arterial assessment by general practitioners, podiatrists, vascular surgeons and nurses to obtain quantitative baseline measures or to confirm diagnosis of arterial insufficiency. Our results might assist health professionals working in wound care within a high risk foot clinic to select and monitor the most appropriate treatments based upon probability of healing in those patients with ulceration. These treatments may involve revascularisation, amputation, or conservative management. Health professionals may also use PPG at baseline and at regular intervals during interventions such as exercise programs, smoking cessation or drug therapies to monitor arterial disease status over time. This would have the added benefit of providing the patient with positive reinforcement that the given intervention is advantageous to their health and importantly having a positive effect on collateral circulation. The Hadeco Smartdop 30EX PPG is an easy to use assessment tool that requires minimal training to perform reliably as an adjunct to clinical examination of the diabetic foot.

Our results should be interpreted within the limitations of the study design. First, the assessors were not blinded to the results of the blood pressures they had taken. Although the assessors were blinded to each other’s results, they were able to see the results of the test they had just completed. However, the Hadeco Smartdop 30EX does not allow for the operator to adjust the result as it is completely automated. To reduce the risk of bias, a print-out of the result was obtained from the machine and all results were given to a research assistant after each examination. Finally, this research focused on the intrarater and interrater reliability of toe pressures and TBI and did not take into account the validity of the test. Although some preliminary research has been conducted in this area, further research is required to determine the validity of these tests compared to standard vascular laboratory tests such as arterial duplex ultrasound and angiography.

## Conclusions

Based on these results, interrater and intrarater reliability of the Hadeco Smartdop 30EX PPG is excellent for measuring toe blood pressure, good for TBI and only fair for brachial pressures in adults with diabetes mellitus.

## Abbreviations

ABI, Ankle brachial index; ICC, Intraclass correlation coefficients; PAD, Peripheral arterial disease; PPG, Photoplethysmography; SEM, Standard error of measurement.

## Competing interests

The research activities of JB are funded by grants from the NHMRC (National Health and Medical Research Council of Australia, Fellowship #1007569 and Centre of Research Excellence #1031893), NIH (National Institutes of Neurological Disorders and Stroke and Office of Rare Diseases, #U54NS065712), Australian Podiatry Education and Research Foundation, Podiatry Council of New South Wales, Charcot Marie Tooth Association, Muscular Dystrophy Association, CMT Association of Australia. The other authors declare that they have no competing interests.

## Authors' contributions

CS participated in the design of the study, secured funding, carried out data collection, contributed to statistical analysis as well as writing and reviewing of the manuscript. KP and LB participated in the design of the study and reviewed the manuscript. DM participated in the design of the study, carried out data collection, and reviewed the manuscript. JB participated in the design of the study, conducted statistical analysis, reviewed the manuscript and provided academic support. All authors reviewed and approved the final manuscript.

## References

[B1] Khan NA Rahim SA Anand SS Simel DL Panju A Does the clinical examination predict lower extremity peripheral arterial disease? JAMA 2006 295 536 546 10.1001/jama.295.5.536 16449619

[B2] de Graaff JC Ubbink DT Legemate DA de Haan RJ Jacobs MJ Interobserver and intraobserver reproducibility of peripheral blood and oxygen pressure measurements in the assessment of lower extremity arterial disease J Vasc Surg 2001 33 1033 1040 10.1067/mva.2001.108011 11331846

[B3] Ramanathan A Conaghan PJ Jenkinson AD Bishop CR Comparison of ankle-brachial pressure index measurements using an automated oscillometric device with the standard Doppler ultrasound technique ANZ J Surg 2003 73 105 108 10.1046/j.1445-2197.2003.02582.x 12608969

[B4] Yamada T Ohta T Ishibashi H Sugimoto I Iwata H Takahashi M Kawanishi J Clinical reliability and utility of skin perfusion pressure measurement in ischemic limbs–comparison with other noninvasive diagnostic methods J Vasc Surg 2008 47 318 323 10.1016/j.jvs.2007.10.045 18241755

[B5] Weatherley BD Chambless LE Heiss G Catellier DJ Ellison CR The reliability of the ankle-brachial index in the Atherosclerosis Risk in Communities (ARIC) study and the NHLBI Family Heart Study (FHS) BMC Cardiovasc Disord 2006 6 7. 10.1186/1471-2261-6-7 16504033 PMC1435775

[B6] Goebel FD Fuessl HS Monckeberg's sclerosis after sympathetic denervation in diabetic and non-diabetic subjects Diabetologia 1983 24 347 350 6873514 10.1007/BF00251822

[B7] Orchard TJ Strandness DE Assessment of peripheral vascular disease in diabetes. Report and recommendations of an international workshop sponsored by the American Heart Association and the American Diabetes Association 18–20 September 1992, New Orleans, Louisiana Diabetes Care 1993 16 1199 1209 8375253 10.2337/diacare.16.8.1199

[B8] Williams DT Harding KG Price P An evaluation of the efficacy of methods used in screening for lower-limb arterial disease in diabetes Diabetes Care 2005 28 2206 2210 10.2337/diacare.28.9.2206 16123491

[B9] Brearley S Shearman CP Simms MH Peripheral pulse palpation: an unreliable physical sign Ann R Coll Surg Engl 1992 74 169 171 1616258 PMC2497570

[B10] Lundin M Wiksten JP Perakyla T Lindfors O Savolainen H Skytta J Lepantalo M Distal pulse palpation: is it reliable? World J Surg 1999 23 252 255 10.1007/PL00013177 9933695

[B11] Alnaeb ME Crabtree VP Boutin A Mikhailidis DP Seifalian AM Hamilton G Prospective assessment of lower-extremity peripheral arterial disease in diabetic patients using a novel automated optical device Angiology 2007 58 579 585 10.1177/0003319707305685 18024941

[B12] Bonham PA Cappuccio M Hulsey T Michel Y Kelechi T Jenkins C Robison J Are ankle and toe brachial indices (ABI-TBI) obtained by a pocket Doppler interchangeable with those obtained by standard laboratory equipment? J Wound Ostomy Continence Nurs 2007 34 35 44 10.1097/00152192-200701000-00007 17228206

[B13] Bonham PA Get the LEAD out: noninvasive assessment for lower extremity arterial disease using ankle brachial index and toe brachial index measurements J Wound Ostomy Continence Nurs 2006 33 30 41 10.1097/00152192-200601000-00004 16444101

[B14] Brooks B Dean R Patel S Wu B Molyneaux L Yue DK TBI or not TBI: that is the question. Is it better to measure toe pressure than ankle pressure in diabetic patients? Diabet Med 2001 18 528 532 10.1046/j.1464-5491.2001.00493.x 11553180

[B15] Romanos M Raspovic A Perrin B The reliability of toe systolic pressure and the toe brachial index in patients with diabetes J Foot Ankle Res 2010 3 31 10.1186/1757-1146-3-31 21176166 PMC3020155

[B16] Burns J Wegener C Begg L Vicaretti M Fletcher J Randomized trial of custom orthoses and footwear on foot pain and plantar pressure in diabetic peripheral arterial disease Diabet Med 2009 26 893 899 10.1111/j.1464-5491.2009.02799.x 19719710

[B17] Enoch A Ijeoma A The Role of Ankle-Brachial Index as a Screening Test for Coronary Artery Disease in the Hispanic Population South Med J 2008 101 1117 1120 10.1097/SMJ.0b013e318189aabc 19088520

[B18] Portney LG Watkins MP Foundations of clinical research: applications to practice Pearson/Prentice Hal 2009 Upper Saddle River, New Jersey 3rd edn

[B19] Fleiss JL Reliability of measurement The design and analysis of clinical experiments 1986 7 John Wiley and Sons, New York

[B20] Allen J Overbeck K Nath AF Murray A Stansby G A prospective comparison of bilateral photoplethysmography versus the ankle-brachial pressure index for detecting and quantifying lower limb peripheral arterial disease J Vasc Surg 2008 47 794 802 10.1016/j.jvs.2007.11.057 18381141

[B21] Carter SA Tate RB Value of toe pulse waves in addition to systolic pressures in the assessment of the severity of peripheral arterial disease and critical limb ischemia J Vasc Surg 1996 24 258 265 10.1016/S0741-5214(96)70101-5 8752037

[B22] Lezack JD Carter SA The relationship of distal systolic pressures to the clinical and angiographic findings in limbs with arterial occlusive disease Scand J Clin Lab Invest Suppl 1973 128 97 101 4764607

[B23] Makisalo H Lepantalo M Halme L Lund T Peltonen S Salmela K Ahonen J Peripheral arterial disease as a predictor of outcome after renal transplantation Transplant Int 1998 11 140 143 10.1007/s001470050446 9664964

